# Infectious diseases in healthcare workers – an analysis of the standardised data set of a German compensation board

**DOI:** 10.1186/1745-6673-7-8

**Published:** 2012-05-03

**Authors:** Albert Nienhaus, Chandrasekharan Kesavachandran, Dana Wendeler, Frank Haamann, Madeleine Dulon

**Affiliations:** 1 Institute for Health Services Research in Dermatology and Nursing, University Medical Centre Hamburg-Eppendorf, Martinistraße 52, 20246, Hamburg, Germany; 2 Institution for Statutory Accident Insurance and Prevention in the Health and Welfare Services, Department of Occupational Health Research, Hamburg, Germany

**Keywords:** Healthcare workers, Infections, Tuberculosis, Needlestick injuries, Blood-borne virus infections

## Abstract

**Introduction:**

Healthcare workers (HCW) are exposed to infectious agents. Disease surveillance is therefore needed in order to foster prevention.

**Methods:**

The data of the compensation board that covers HCWs of non-governmental healthcare providers in Germany was analysed for a five-year period. For hepatitis B virus (HBV) and hepatitis C virus (HCV) infections, the period analysed was extended to the last 15 years. The annual rate of occupational infectious diseases (OIDs) per 100,000 employees was calculated. For needlestick injuries (NSI) a rate per 1,000 employees was calculated.

**Results:**

Within the five years from 2005 to 2009 a total of 384 HCV infections were recognised as OIDs (1.5/100,000 employees). Active TB was the second most frequent cause of an OID. While the numbers of HBV and HCV infections decreased, the numbers for active TB did not follow a clear pattern. Needlestick injuries (NSIs) were reported especially often at hospitals (29.9/1,000 versus 7.4/1,000 employees for all other HCWs).

**Conclusion:**

Although they are declining, HCV infections remain frequent in HCWs, as do NSIs. Whether the reinforcement of the recommendations for the use of safety devices in Germany will prevent NSIs and therefore HCV infections should be closely observed.

## Introduction

It is well known that healthcare workers (HCWs) risk contracting infectious diseases [1]. HCWs are at risk of infection with blood-borne pathogens during occupational exposure to blood and body fluids [2]. The increased risk of tuberculosis (TB) infection for HCWs is well documented [3-5]. Emerging infectious diseases such as severe acute respiratory syndrome (SARS), H5/N1 avian influenza and H1/N1 swine influenza have shown the particular vulnerability of HCWs. SARS was identified as a new disease by WHO physician Dr. Carlo Urbani. He diagnosed it in a patient who died from the illness. Dr. Urbani subsequently died from SARS, too. SARS affected 8,096 individuals globally, 21% of whom were HCWs [6,7]. SARS has been known to spread extensively among HCWs in various settings. In outbreaks in Hong Kong and Toronto, 62% [8] and 51% [9] of the infected patients were HCWs.

During the first outbreak of H5/N1 avian infections in Hong Kong in 1997, the prevalence of H5/N1 antibodies was five times higher in exposed HCWs than in HCWs without contact to avian influenza patients, i.e. 3.7% (8/217) versus 0.7% (2/309) [10]. Even though H1/N1 swine influenza was well contained in Portugal, HCWs were at increased risk of contracting this infection [11]. The increased risk of infection for HCWs is not always easy to detect. Working in healthcare was for a long time considered a safe place offering protection against tuberculosis. Only when the prevalence of tuberculosis declined in the general population did it become apparent that the rate of latent tuberculosis infection (LTBI) and active tuberculosis was high in those caring for tuberculosis patients [12]. With the further decline of tuberculosis in high-income countries, interest in tuberculosis as an occupational disease dwindled. It was only with the emergence of HIV that interest in tuberculosis as a co-infection inspired research and fostered the prevention of infectious diseases in HCWs. In the aftermath of the HIV emergence, awareness of accidental blood contact and needlestick injuries rose, leading finally to the Needlestick Safety and Prevention Act in the US [13] and to the mandatory introduction of safety devices in Germany (TRBA 250 [14]) in order to prevent needlestick and sharps injuries as well as blood contact between patients and HCWs.

Surveillance data on occupational health risks is a cornerstone in occupational safety and health (OSH) management. Therefore new members of the enlarged European Union (EU) were encouraged to adopt the EU’s list of occupational diseases (ODs) and to build up their own monitoring system for surveying the burden of ODs in their countries [15]. Reporting the data on claims of occupational infectious diseases (OIDs) filed by HCWs with the compensation board not only sheds light on important time trends in work-related health risks, but also stresses the importance of workplace prevention and hygiene. This type of surveillance system can be adopted by other countries with evolving social security systems in order to reduce the disease burden in HCWs [16].

Even though hygiene standards are high and access to vaccination is comprehensive in high-income countries like Germany, HCWs still remain vulnerable to infections at their workplaces. The aim of our study was to describe the number of infectious diseases in HCWs and to describe time trends using the standardised database of a compensation board.

## Methods

The *Berufsgenossenschaft* for the healthcare (Gesundheitswesen) and welfare services (BGW) (Institution for Statutory Accident Insurance and Prevention) is the compensation board for all non-governmental healthcare and welfare providers in Germany. A total of 500,000 enterprises with about six million paid workers are covered by the BGW. The BGW’s database on compensation claims concerning the occupational disease number 3101, “infectious disease with human-to-human transmission”, was used for this analysis. The database allows a distinction to be made between the most frequent infectious diseases. Furthermore accidental blood contact reported to the compensation board can be identified. Contact with blood (or other body fluids) splashed onto the skin or the mucous membranes is coded as accidental blood contact (ABC). No information is available on whether the ABC occurred on intact or non-intact skin or on skin or mucous membranes. Injuries caused by used needles, scalpels or knives are coded as needlestick injuries (NSIs).

The reporting of suspected cases of ODs is compulsory for physicians and companies after first diagnosis. In the standardised data set, a further distinction is made between ODs that are mandatorily reportable and those that are not. Reporting ABC is not mandatory. ABC in the form of splashes is documented as an OID. It is reported to the compensation board because the cost of post-exposure prevention is covered by the board. NSIs are documented as working accidents. They must only be reported if they cause sick leave of more than three days, which they do not normally. However, they are reported in order to obtain compensation for the cost of post-exposure prevention independent of any sick leave.

The distinction between infectious diseases was introduced at different times and is available only for the most important infectious diseases. Claims of OIDs were analysed for 2009. Time trends over five years were analysed for claims concerning tuberculosis (TB), latent tuberculosis infection (LTBI), scabies, methicillin-resistant Staphylococcus aureus (MRSA), human immunodeficiency virus/acquired immunodeficiency syndrome (HIV/AIDS), ABC and NSIs. As the data set allows a distinction to be made between hepatitis B virus (HBV) and hepatitis C virus (HCV) starting in 1995, time trends are given for the 15 years between 1995 and 2009 for these infections. LTBI was considered present if the interferon-γ release assay (IGRA) was positive and active TB was excluded by X-ray.

We calculated rates of OIDs per 100,000 employees and of NSIs and ABC per 1,000 employees. In order to reduce variation by chance, these rates were calculated as an average for the last five years.

The prevalence of OIDs, ABCs and NSI were analysed for selected working areas in healthcare. These working areas correspond to clustered occupational groups, including settings with comparable risks of accidents and diseases, which are used in combination with compensation money to calculate insurance premiums. For the analyses, four working areas were selected: 1.) hospitals and other clinical facilities (referred to as hospitals in the following), 2.) medical surgeries with all kind of specialisations (referred to as surgeries), 3.) nursing homes, hospices, and other long-term care facilities (referred to as nursing homes), 4.) outpatient medical and social care, emergency medical services (referred to as outpatient care). The remaining occupational groups – including veterinary practices, hairdressing, administration, day care, social welfare services and facilities for the handicapped – were pooled (referred to as others in the following). Five-year trends for the most frequent infectious diseases were analysed separately for these working areas. Regulations for using safety devices were bolstered in Germany in 2008. Therefore claims of NSIs and ABC were analysed depending on working areas for 2008 and 2009 in order to see whether the new regulations had decreased the number of NSIs or ABC in the different working areas. Costs for NSIs and ABC were extracted from the data set for a five-year period.

## Results

**Figure 1:**
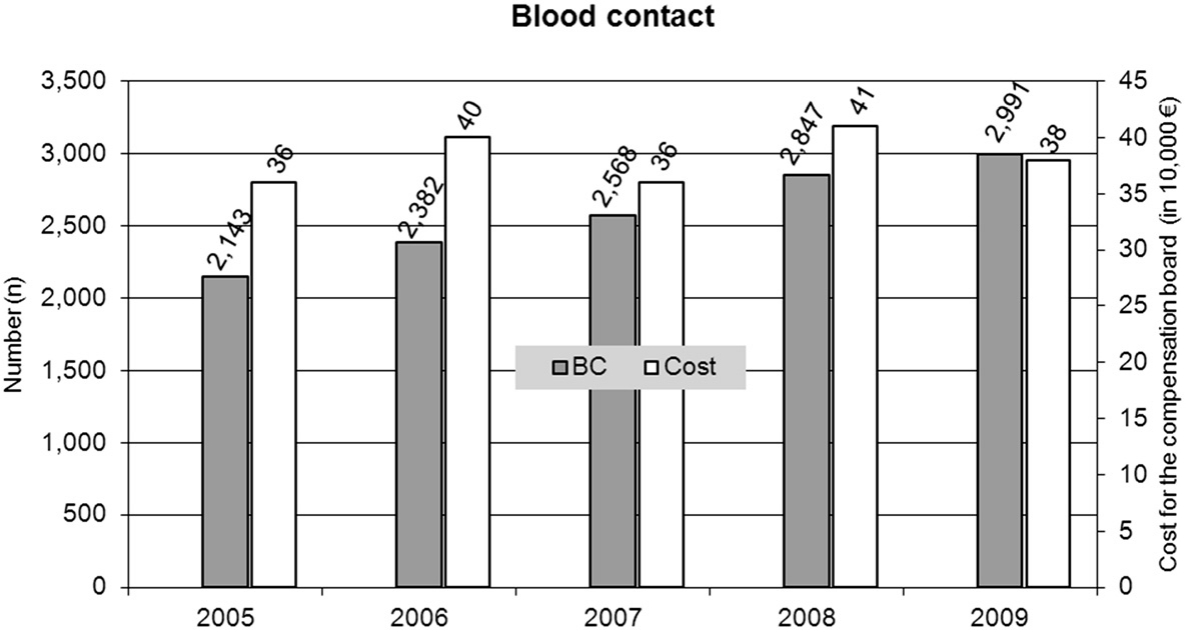
Number of claims of blood contact (BC) and cost for the compensation board from 2005 to 2009; data from BGW, Germany.

**Figure 2:**
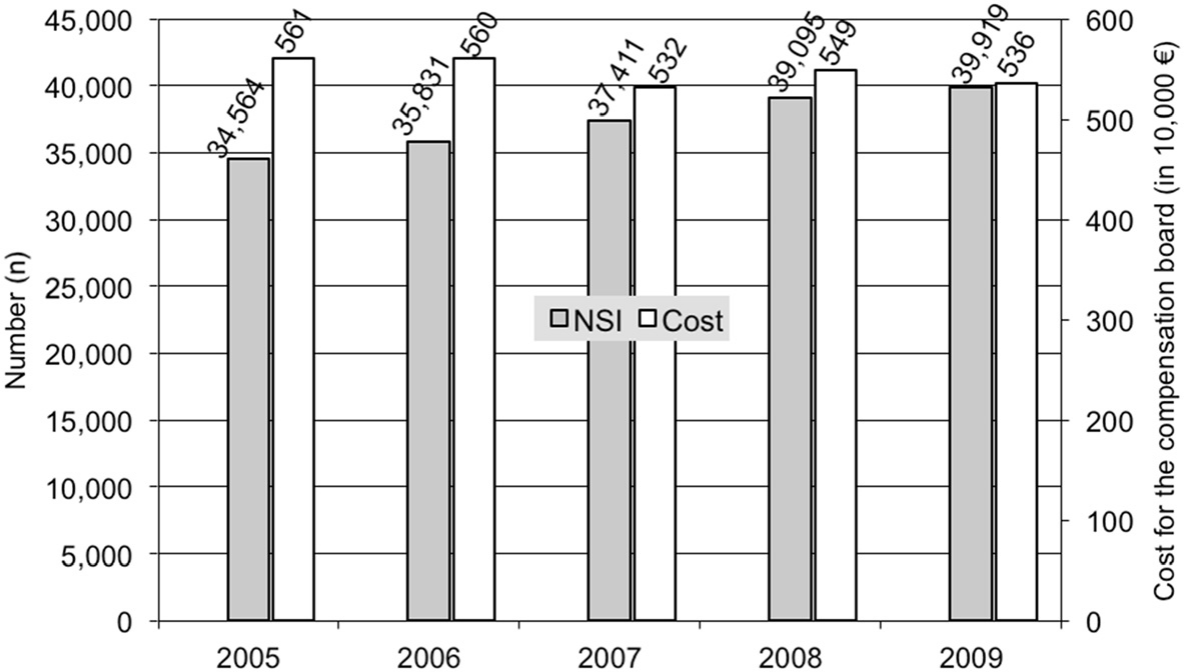
Number of claims of needlestick injuries (NSIs) and cost for the compensation board from 2005 to 2009; data from BGW, Germany.

A total of 3,008 infections were claimed in 2009 (Table 1). The bulk of them were not mandatorily reportable (69%). Scabies was the most frequent cause of a claim (38%). However, most of these claims were not reportable contacts (90%) and not actual infections with scabies. The second most frequent cause of a claim was tuberculosis (26%). Again, most often these claims concerned notifications of contact with infectious patients or materials (60%) and were therefore not reportable. Influenza (9%) and MRSA (8%) followed thereafter, with less than 10% of the claims. Again, most claims concerned infectious contact rather than actual infections. Ten percent of all claims were claims due to hepatitis virus infections. However, two-thirds of these claims were mandatory (62%). HCV was the most frequent claim here, followed by HBV. Less than 1% of the claims related to HIV/AIDS. In addition to the blood-borne virus infection claims, in 2009 a total of 2,991 cases of ABC and 39,919 NSIs were reported (Figures 1 and 2).

**Table 1:** Number of suspected cases of occupational infectious diseases (OD no. 3101) for 2009 separated by notification requirement, data from BGW, German

**Occupational infectious diseases (OD no. 3101)**	**Mandatory**	**Not mandatory**	**Total(col %)**
	**N (row %)**	**N (row %)**	
Tuberculosis (TB)	311 (40)	472 (60)	783 (26)
Hepatitis virus infection (total)	181 (62)	113 (38)	294 (10)
Thereof A	4 (57)	3 (43)	7 (<1)
B	75 (60)	50 (40)	125 (4)
C	101 (64)	58 (36)	159 (5)
D	0	0	0
E	1 (33)	2 (67)	3 (<1)
Scabies	110 (10)	1,026 (90)	1,136 (38)
MRSA/ORSA	102 (44)	128 (56)	230 (8)
Influenza	53 (21)	206 (79)	259 (9)
Angina/pertussis	27 (100)	0	27 (1)
Keratoconjunctivitis	23 (92)	2 (8)	25 (1)
HIV/AIDS	5 (33)	10 (67)	15 (<1)
Other	125 (52)	114 (48)	239 (8)
**Total**	**937 (31%)**	**2,071 (69%)**	**3,008**

**Figure 3:**
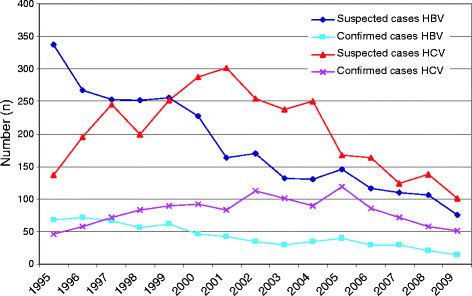
Number of suspected and recognised cases of HBV and HCV infections from 2005 to 2009, data from BGW, Germany.

Over the last 15 years the number of reportable claims of suspected OIDs has declined along with the number of HBV and HCV infections recognised as OIDs (Figure 3). While claims for HCV infections increased from 1995 to 2002, followed by a sharp decrease thereafter, a continuous decrease for HBV infections was observed starting in 1995. The number of recognised HCV cases only started to decline after 2005, while for HBV a decrease was seen over the whole period again. These temporal trends are not confirmed by reports of ABC or NSIs. Reports of ABC increased from 2,143 in 2005 to 2,991 in 2009 (Figure 1). Reports of NSIs increased from 2005 until 2009 by 2% to 5% annually (Figure 2, Table 2). In 2009, the cost for the compensation board amounted to 380 thousand euros for ABC and 5.36 million euros for NSIs. The cost of ABC and NSI did not increase proportionally to the increase of claims. Reasons for these disproportional changes are unknown.

**Table 2:** Number of needlestick injuries and blood contact cases and annual mean rates per 1,000 employees by different risk groups in 2008 and 2009; data from BGW, Germany

	**Needlestick injuries**				**Blood contact**			
	2008	2009			2008	2009		
**Risk group**	**N(col%)**	**N(col%)**	Difference^*^(%)	**Annual rate**^+^	**N(col%)**	**N(col%)**	Difference^*^(%)	**Annual rate**^**+**^
Hospitals	21,896 (56)	22,100 (55)	+0,9	29.9	1,996 (70)	2,089 (70)	+4.7	2.8
Surgeries	8,379 (21)	8,442 (21)	+0.8	12.0	413 (15)	464 (16)	+12.3	0.6
Nursing homes	3,828 (10)	4,073 (10)	+6.4	3.6	139 (5)	168 (6)	+20.9	0.1
Outpatient care	1,589 (4)	1,706 (4)	+7.3	4.2	46 (2)	41 (1)	-10.9	0.1
Others	3,403 (9)	3,598 (9)	+5.7	1.5	253 (9)	229 (8)	-9.4	0.1
**Total**	**39,095**	**39,919**	**+2.1**	**7.4**	**2,847**	**2,991**	**+6.7**	**0.6**

* Difference calculated as change from 2008 to 2009; negative change means reduction; positive change means increase.

^+^ Calculated per 1,000 employees averaged over two years (2008 and 2009).

Even though suspected and recognised cases of HCV infection have declined over the last five years (Table 3), the average OID rate per 100,000 employees was highest for this particular infectious disease (1.5/100,000 employees). Scabies and active TB also have similar OID rates. For scabies the number of recognised OIDs declined, being highest in 2005. No clear pattern was seen for active TB. However, for LTBI recognised as an OID, the absolute number increased from five in 2006 to 65 in 2009. In 2005 the data set did not yet allow for a distinction to be made between LTBI and active TB.

**Table 3:** Number of suspected (S) and recognised (R) cases of selected occupational infectious diseases by year of notification and annual rates of recognised cases per 100,000 employees; 2005−2009; data from BGW, Germany

**Occupational infectious diseases**		**Year**					**Total**	**Annual rate of recognised cases**^**+**^
		**2005**	**2006**	**2007**	**2008**	**2009**		
		**N**	**N**	**N**	**N**	**N**	N (%*)	
Active TB	S	234	175	253	195	124	990	
	R	99	83	56	73	61	372 (38)	1.5
LTBI	S	-	20	33	40	187	280	
	R	-	5	6	17	65	93 (33)	0.4
Scabies	S	-	701	355	135	110	1,301	
	R	-	128	88	102	58	376 (29)	1.5
MRSA	S	-	114	88	98	102	402	
	R	-	1	5	11	8	25 (6)	0.1
HBV	S	146	116	110	106	75	553	
	R	40	29	30	20	14	133 (24)	0.5
HCV	S	168	163	124	138	101	694	
	R	119	86	71	57	51	384 (55)	1.5
HIV/AIDS	S	9	12	4	11	5	41	
	R	2	0	3	1	0	6 (15)	0.02

* Proportion of the suspected cases that were recognised as occupational infectious diseases between 2005 and 2009.

^+^ Calculated per 100,000 employees averaged over five years (2005–2009).

LTBI = latent TB infection.

ABC (55%) and NSIs (70%) are most often reported at hospitals (Table 2). In 2009, the rate per 1,000 employees in hospitals was 29.9 for NSIs and 2.8 for ABC. The second highest NSI rate per 1,000 employees was seen in surgeries (12.0). Claims of NSI increased by 2.1% from 2008 to 2009, with the largest rise seen in outpatient care (7.3%). Claims of ABC increased by 6.7% from 2008 to 2009, with the largest rise seen in nursing homes (20.9%). The rate for all recognised OIDs was highest in hospitals and second highest in nursing homes (15.3 versus 8.3/100,000, Table 4). In hospital workers the annual mean rate of recognised OIDs was highest for TB (5.7/100,000) followed by HCV (4.8/100,000).

**Table 4:** Annual mean rates of recognised cases of occupational infectious diseases per 100,000 employees by different risk groups; 2005−2009; data from BGW, Germany

**Occupational infectious disease**	**Risk group**					**Total**
	**Hospitals**	**Surgeries**	**Nursing homes**	**Outpatient care**	**Others**	
Active TB	5.7	2.1	0.8	0.8	0.4	1.5
LTBI	1.1	0.7	0.2	0.2	0.1	0.4
Scabies	1.3	0.1	6.0	0.6	0.1	1.5
MRSA	0.3	0.0	0.3	0.2	0.0	0.1
HBV	1.9	0.7	0.2	0.3	0.2	0.5
HCV	4.8	3.0	0.8	1.4	0.5	1.5
HIV/AIDS	0.1	0.0	0.0	0.0	0.0	0.02
**Total**	**15.3**	**6.6**	**8.3**	**3.5**	**1.3**	**4.5**

LTBI = latent TB infection.

## Discussion

To our knowledge this study is the first attempt to describe in detail the number of infectious diseases in German HCWs using data of a compensation board. This analysis has revealed different time trends. The most significant trend is that the number of HBV and HCV infections recognised as OIDs has declined during the last 15 years, with HCV infections now more often being the cause of an OID than HBV infections. However, the cases of NSIs and ABCs (splashes) filled to the board increased steadily over the last five years.

So far, only a few analyses of compensation claims by HCWs have been published. A US analysis of NSI claims by HCWs eligible to file a state fund workers’ compensation claim in Washington State established that the incidence of NSI claims per 1,000 HCWs employed in hospitals was 15.9, in dental offices 10.5, in physicians’ offices 8.7 and in skilled nursing facilities 8.1 [17]. In our data set, rates of NSIs per 1,000 employees were twice as high in hospitals (29.8) but half as high in nursing homes (3.7). However, not all facilities clustered in this working area can be classified as skilled nursing facilities and therefore our denominator in this group is inflated. In addition comparison of the two studies is not possible as different time periods were analysed (1996 to 2000 in the US study [17] and 2008 to 2009 in our study).

A second study based on compensation claims for OIDs was a case series of HCWs with confirmed MRSA infections [18]. The authors used the same data set as we did to identify MRSA-related OID claims and performed an in-depth analysis of the individual files. In total 17 MRSA-related OIDs were identified in HCWs. The analysis of compensation claims by veterinarians in Germany found an increased risk of animal-related accidents and infections [19]. The literature that has been published to date on claims filed by HCWs makes it clear that, whatever the shortcomings of this data, analysing compensation claims by HCWs can be helpful in tackling the occupational risks faced by HCWs.

In Germany, a low-incidence country for TB, the rate of active TB as an OID in HCWs was even lower than expected (5.5/100,000 in the general population and 1.5/100,000 in HCWs). Under-representation of HCWs among patients with active TB was also observed in the Hamburg fingerprint study [5]. However, it should be noted that HCWs do not share most other risks for active TB, for example homelessness, intravenous drug abuse or alcoholism. Therefore the risk of progression to active TB seems to be lower in HCWs than in close contacts of the general population [11]. LTBI was reported and recognised as an OID more often in 2008 and 2009 than in the years before. This reflects the improvement in diagnosing LTBI, which was brought about by introducing the interferon-γ release assay into routine screening of HCWs [20-24]. High progression rates in close contacts to TB patients observed in Germany further fostered the awareness of LTBI as a risk for HCWs [25,26].

HBV infection can be prevented by vaccination. In Germany, vaccinations are offered to every HCW at risk of infection during a mandatory physical examination at the workplace following the Biological Agents Ordinance. Therefore, HBV vaccination coverage in German HCWs is high and the decreasing number of HBV-related OIDs is well explained. However, the number of HCV-related OIDs decreased as well. A similar trend was observed in France [2]. As no vaccine is available for HCV, this might indicate improvements in the realm of occupational hygiene.

Experiences in the US and Francedemonstrate that NSIs can be prevented [13,27]. In Germany the number of NSIs reported is still high, and it should be assumed that the real incidence of NSIs in HCWs is even higher due to under-reporting. In 2008 regulations on the use of safety devices became more rigorous in Germany. Following these regulatory changes, safety devices were more often used in German healthcare. In the absence of a systematic evaluation, the experience in Hamburg might be reported. After informing hospitals about the new regulations and advising these hospitals on safety devices, all hospitals in Hamburg introduced them to varying degrees and subsequently reported a decrease in NSIs [28]. However, the number of NSI related claims to the compensation board so far did not decrease. Annual costs for ABC and NSIs amounted to about 4 million euros. However, it should be noted that the real cost is underestimated because the compensation cost for blood-borne infections is not included in this estimate and under-reporting of ABC and NSIs is very likely. A more in-depth analysis of the cost of OIDs might be helpful in order to demonstrate the economic burden of OIDs in healthcare.

## Limitations

This analysis was based on the routine data of one compensation board, which covers about two thirds of all HCWs in Germany. The completeness of the data depends on the willingness of physicians and HCWs to report incidents. It might be safe to assume that – especially with less severe infectious diseases – there is considerable under-reporting because little monetary interest is involved. The annual incidence of NSI in HCWs of a university hospital was 31.4% [29]. Compared to the annual rate of 29.9 NSI per 1,000 employees in hospitals (Table 2), it follows that about 10% of the NSI are reported to the compensation board. The advisors who are responsible for documenting claims are trained for their task. However, the quality of data entries is controlled in a rather superficial way. Therefore misclassification is likely. These limitations considered, the standard data set on occupational diseases of the *Berufsgenossenschaft* (compensation board) allow for a cautious estimate of the risk of infectious diseases in HCWs in Germany and enable meaningful time trends to be observed.

As the risk for OID remains high (e.g. 15.3/100,000 hospital employees, Table 4), awareness for the infection risk and knowledge about infection prevention should be improved [30,31]. Communicating the safety and effectiveness of vaccination will be an important issue in this endeavour [32-34] as well as improvements in protective equipment likes gloves or safety devices [35,36].

## Conclusion

Even though Germany is a low-incidence country, TB still poses a threat for HCWs and screening for TB should therefore be maintained. Even though they are declining, HBV and HBV infections are still frequent and trends should be watched closely. Trends in NSIs should be observed closely in the following years in order to evaluate the effect of new regulations on the use of safety devices.

## Competing interest

The authors work for the compensation board for which the data were analysed. However, the compensation board did not try to influence the content of this article in any way. Therefore, the authors declare that they do not have any direct or indirect personal relationship, affiliation or association with any party with whom they deal in their day-to-day work that would give rise to any actual or perceived conflict of interest.

## Authors’ contributions

AN wrote the paper. CK made substantial contributions to the revision of the first draft. DW analysed the data. FH made substantial contributions to the revision of the first draft. MD analysed the data and made substantial contributions to the revision of the first draft. All authors read and approved the final manuscript.
